# Serum proteomic biomarker investigation of vascular depression using data-independent acquisition: a pilot study

**DOI:** 10.3389/fnagi.2024.1341374

**Published:** 2024-02-07

**Authors:** Liuyi Lan, Sisi Peng, Ran Zhang, Haoying He, Yong Yang, Bing Xi, Junjian Zhang

**Affiliations:** ^1^Department of Neurology, Zhongnan Hospital, Wuhan University, Wuhan, China; ^2^Department of Neuropsychology, Zhongnan Hospital of Wuhan University, Wuhan, China; ^3^SpecAlly Life Technology Co., Ltd., Wuhan, China

**Keywords:** vascular depression (VaD), serum proteome, biomarkers, cerebrovascular disease, machine learning

## Abstract

**Background:**

Vascular depression (VaD) is a depressive disorder closely associated with cerebrovascular disease and vascular risk factors. It remains underestimated owing to challenging diagnostics and limited information regarding the pathophysiological mechanisms of VaD. The purpose of this study was to analyze the proteomic signatures and identify the potential biomarkers with diagnostic significance in VaD.

**Methods:**

Deep profiling of the serum proteome of 35 patients with VaD and 36 controls was performed using liquid chromatography–tandem mass spectrometry (LC–MS/MS). Functional enrichment analysis of the quantified proteins was based on Gene Ontology (GO), Kyoto Encyclopedia of Genes and Genomes (KEGG) pathway, and Reactome databases. Machine learning algorithms were used to screen candidate proteins and develop a protein-based model to effectively distinguish patients with VaD.

**Results:**

There were 29 up-regulated and 31 down-regulated proteins in the VaD group compared to the controls (|log_2_FC| ≥ 0.26, *p* ≤ 0.05). Enrichment pathways analyses showed that neurobiological processes related to synaptic vesicle cycle and axon guidance may be dysregulated in VaD. Extrinsic component of synaptic vesicle membrane was the most enriched term in the cellular components (CC) terms. 19 candidate proteins were filtered for further modeling. A nomogram was developed with the combination of HECT domain E3 ubiquitin protein ligase 3 (HECTD3), Nidogen-2 (NID2), FTO alpha-ketoglutarate-dependent dioxygenase (FTO), Golgi membrane protein 1 (GOLM1), and N-acetylneuraminate lyase (NPL), which could be used to predict VaD risk with favorable efficacy.

**Conclusion:**

This study offers a comprehensive and integrated view of serum proteomics and contributes to a valuable proteomics-based diagnostic model for VaD.

## 1 Introduction

Vascular depression (VaD) is a depressive syndrome occurring in individuals aged 50 years and older and is associated with cerebrovascular comorbidities and vascular risk factors (VRFs). It is characterized by the presence of deep white matter hyperintensity (DWMH) on T2-weighted brain magnetic resonance imaging (MRI) (Alexopoulos et al., 1997; Krishnan et al., 2004; Sneed et al., 2008). It is considered a distinct subtype of late-life depression (LLD), accounting for approximately 54% of LLD cases, with a prevalence of approximately 3.4%. It is more prevalent in middle-aged and older adult patients with cerebrovascular diseases (González et al., 2012; Park et al., 2015). Importantly, individuals with VaD often poorly respond to antidepressant therapy and exhibit more cognitive impairment (especially executive function decline) than those with depression without vascular risk factors (Steffens, 2019). Vascular depression is considered a clinical risk factor for dementia, and recent research shows that mild behavioral abnormalities occur earlier in people with dementia than in those with mild cognitive disabilities (Van der Mussele et al., 2013; Matsuoka et al., 2019).

Vascular depression pathogenesis is multifaceted and involves biological and psychological factors. Ischemic cerebrovascular processes significantly contribute to severe DWMH in LLD (Thomas et al., 2002, 2003). The disruptions in white matter connectivity can result in cognitive impairments and depressive symptoms, supporting the “vascular depression hypothesis” (van Sloten et al., 2015; van Agtmaal et al., 2017; Geraets et al., 2020). In addition to cerebrovascular processes, the underlying mechanisms of VaD include neuroinflammation, oxidative stress, neurovascular dysfunction, and neurotransmitter imbalance (Strawbridge et al., 2017; Jellinger, 2022).

Due to the diversity of symptoms and the complexity of the pathogenesis, the diagnosis of VaD is difficult with a lack of reliable biomarkers. Several studies have explored the biomarkers for depression via peripheral blood-based proteins and have mainly focused on five systems, involving inflammation (IL-6, CRP), neurotransmitter components (serotonin 1A receptor), neuroendocrine (cortisol), neurotrophic (brain-derived neurotrophic factor, BDNF) and metabolic processes (Kennis et al., 2020; Malik et al., 2021). However, there is a significant disparity among the findings. The utilization of machine learning algorithms based on large proteomic data may be beneficial to address the challenges of biomarker heterogeneity and variability and to identify the optimal biomarkers in depression (Guo et al., 2022). When the low sensitivity and specificity of a single biomarker and the interactions between biomarkers are taken into account, the multiprotein panels were proposed to improve clinical diagnosis practice and better describe the complexity of disease phenotypes (Schmidt et al., 2011).

Non-targeted proteomic methods based on liquid chromatography–tandem mass spectrometry (LC–MS/MS) are powerful tools for investigating potential biomarkers. In contrast to traditional data acquisition, data-independent acquisition (DIA) systematically and repeatedly separates and fragments the m/z range, enabling in-depth protein profiling with low sample requirements in an unbiased manner (Demichev et al., 2020).

This study conducted a comprehensive proteomic analysis of serum samples from VaD and control patients to identify the differential proteins and biological pathways and to develop a promising protein-based model with diagnostic significance for VaD using machine learning methods.

## 2 Materials and methods

### 2.1 Participants

We used a convenience sample of 71 right-handed participants aged 55–75 years, including 35 patients with VaD and 36 non-depressive individuals (controls). The participants were part of an ongoing cohort study conducted in the Department of Neurology, Zhongnan Hospital of Wuhan University. Two trained clinicians performed the diagnosis of VaD by referring to criteria from the consensus report (Aizenstein et al., 2016), including (1) having any depressive disorder type as defined in the Diagnostic and Statistical Manual of Mental Disorders, fifth edition (DSM-V); (2) having the cerebrovascular disease; (3) with at least one of the VRFs (including smoking, hypertension, diabetes mellitus [DM], cardiovascular disease, and hyperlipidemia); and (4) with no suspicious depressive episodes preceding obvious cerebrovascular disease. The criteria for inclusion in the VaD group were: (1) meeting the diagnosis standard of VaD; (2) presence of severe DWMH in MRI, which was defined as Fazekas score ≥ 2; (3) depressive behavioral symptoms lasting over 2 weeks; (4) not using antidepressants or antipsychotics for at least 3 months; and (5) with Mini-Mental State Exam (MMSE, Beijing version) scores ≥17 for illiteracy, ≥ 20 for individuals with 1–6 years of education, and ≥ 24 for individuals with 7 or more years of education. The criteria for inclusion in the control group were: no history of major depressive disorder (MDD) or any other mental disorders; and no mild cognitive impairment (Li et al., 2016).

The exclusion criteria were as follows: (1) WMH owing to non-vascular dysfunction; (2) stroke history in 6 months; (3) recent life events; (4) a severe physical disability; (5) comorbid neurodegenerative diseases (such as Parkinson’s disease or Alzheimer’s disease) or other acute, severe, or unstable medical conditions; and (6) hearing and comprehension dysfunction and inability to cooperate with neuropsychological assessments.

This study was approved by the institutional ethics committee of Zhongnan Hospital, Wuhan University (2,020,124/2023133 K). For all participants, written informed consent was obtained. The Declaration of Helsinki was followed in the conduct of the study.

### 2.2 Clinical and neuropsychological assessments

We collected data on clinical and demographic characteristics, including age, sex, education, BMI, and VRFs. Medical records established the presence of VRFs, which were either diagnosed by a physician or self-reported. An experienced psychologist performed the neuropsychological assessments of all participants. The Hamilton Depression Scale 17-items (HAMD-17) and Hamilton Anxiety Scale 14-items (HAMA-14) were used to evaluate depressive and anxiety symptoms, respectively (Goldberger et al., 2011). MMSE was applied to assess cognitive function and dementia screening (Li et al., 2016). The Trail Making Test consisted of two parts evaluated separately (A and B). Part A evaluated psychomotor speed, and Part B assessed visual and spatial working memory and cognitive flexibility (Wei et al., 2018).

### 2.3 Magnetic resonance imaging evaluation

Magnetic resonance images were obtained with a 3.0 T MR scanner (Siemens Healthcare, Erlangen, Germany). The FLAIR sequence parameters were as follows: repetition time (TR) = 6,000 ms, echo time (TE) = 388 ms; echo train length = 848, bandwidth = 781 Hz/pixel; voxel size, 1 × 1 × 1 mm; field of view, 256 × 256 mm; and 176 sagittal slices. The WMHs were evaluated using the visual rating scale (Fazekas et al., 1988). Periventral hyperintensities (PVH) and DWMH were rated separately by two radiologists.

### 2.4 Proteomic analysis

#### 2.4.1 Sample preparation

The samples were enriched using superparamagnetic iron oxide nanoparticles. Twenty μl of the sample was diluted with loading buffer (10 mM Tris-Cl, 1 mM EDTA, 150 mM KCl, 0.05% CHAPS) and mixed with 1 mg of magnetic beads. The mixture was incubated at 37°C for 1 h. The beads were washed twice with loading buffer and then once with CHAPS-free buffer (10 mM Tris-Cl, 1 mM EDTA, 150 mM KCl). The magnetic beads were collected on a magnetic rack and the supernatant was discarded to obtain the protein-rich magnetic beads. The sample was then added with lysis buffer (1% SDC/100 mM Tris–HCl, pH = 8.5/10 mM TCEP/40 mM CAA) and incubated at 60°C for 30 min for protein reduction and alkylation. An equal volume of ddH2O was added to dilute the SDC to a concentration below 0.5%, and 1 μg of trypsin was added. The mixture was incubated overnight at 37°C for enzymatic digestion. The next day, the pH was lowered to 6.0 with TFA to complete the digestion. After centrifugation, the supernatant was subjected to peptide purification using a home-made SDB-RPS desalting column. The peptide eluate was vacuum dried and stored at −20°C for later use.

#### 2.4.2 LC–MS/MS analysis

All samples were analyzed on timsTOF Pro (Bruker Daltonics). An UltiMate 3,000 RSLCnano system (Thermo) was coupled to timsTOF Pro with a CaptiveSpray nano ion source (Bruker Daltonics). Peptides were injected into a C18 Trap column with dimensions of 75 μm by 2 cm, consisting of particles that were 3 μm in size and 100 Å pore size from Thermo. They were subsequently separated in a reversed-phase C18 analytical column that measured 75 μm by 25 cm, comprising particles that were 1.6 μm in size and 100 Å pore size from IonOpticks. Mobile phase A (0.1% formic acid in water) and mobile phase B (0.1% formic acid in ACN) were utilised to establish a separation gradient lasting 60 min. This gradient began with a mixture of 6 to 11% B within 5 min, followed by a gradual increase to 25% B within 35 min, leading to a further rise to 50% B in 15 min, then ending with a 3-min wash at 90% B and a 2-min re-equilibration at 6% B. Meanwhile, a flow rate of 300 nL/min was maintained. The MS was operated in diaPASEF mode (Meier et al., 2020). The capillary voltage was set to 1,400 V, and the MS and MS/MS spectra were obtained from 100 to 1700 m/z. The ion mobility was scanned from 0.6 to 1.6 *Vs*/cm2. The accumulation and ramp times were 100 ms. The timsControl software (Bruker Daltonics) was used to define the diaPASEF acquisition scheme based on the m/z-ion mobility plane. The collision energy was linearly ramped with mobility, from 59 eV at 1/K0 = 1.6 *Vs*/cm2 to 20 eV at 1/K0 = 0.6 *Vs*/cm2.

#### 2.4.3 Proteomics preprocessing

The library-free mode of DIA-NN (V1.8.1) was used to analyze DIA raw data (Demichev et al., 2020). Spectra files were searched against the sequence database downloaded from UniProt (The UniProt Consortium, 2023). Search parameters were set to default with the following modifications: Precursor ion generation options were enabled for *in silico*-predicted spectral library generation; Trypsin/P with a maximum of two missed cleavages was used; Carbamidomethyl on C was applied as a fixed modification; Oxidation on M and N-terminal acetylation were used as variable modifications for proteins. Mass and MS1 accuracy were adjusted to 15 ppm, MBR was enabled, and Heuristic protein inference was used. For reliable identifications, Precursor FDR was set at 1%. MaxLFQ algorithm was used to normalize protein intensities (Cox et al., 2014).

#### 2.4.4 Biological analysis

Statistical significance was assessed by unpaired t-test to identify the differentially expressed proteins (DEPs). Proteins with *p* < 0.05, fold change (FC) > 1.2, or < 1/1.2 were considered significantly changed. Functional enrichment analysis of quantified proteins was based on Gene Ontology (GO),^1^ Kyoto Encyclopedia of Genes and Genomes (KEGG),^2^ and Reactome database^3^ (Gillespie et al., 2022). CytoScape software and the “CytoHubba” plug-in were used to establish a protein–protein interaction (PPI) network of DEPs based on the STRING database (Shannon et al., 2003).

### 2.5 Statistical analysis

Depending on the distribution of the data, the differences between the two groups were compared by the following tests: two-sample *t*-test, χ^2^ test, or Mann–Whitney U test. Partial correlation analysis was performed after controlling for age, sex, education, BMI, smoking, hypertension, DM, cardiovascular disease, hyperlipidemia, PVH, and DWMH. The extreme gradient boosting (XGBoost) method and the least absolute shrinkage and selection operator (LASSO) regression were used to screen protein features. Proteins were sorted by importance score (gain percent). The protein features with gain percentages greater than zero were included for subsequent analysis. Following 10-fold cross-validation, the parameters for LASSO regression analysis with the smallest model fitting error were utilized to further filter the variables. Logistic regression (LR), a widely used machine learning algorithm, was performed for model training and parameter optimization. For modeling data processing, it was proposed that 75% of the data was used as a training set and 25% of the data as a testing set. The best model with diagnostic accuracy was found by calculating the area under the curve (AUC) using receiver operating characteristic (ROC) curves (de Hond et al., 2022). The calibration curve was used to evaluate the clinical prediction model, and the clinical effect of the model was assessed by clinical decision curve analysis. A nomogram was further conducted to predict the risk of VaD (Alba et al., 2017). Statistical analyses and model formulation were performed using R software (version 4.2.0) or Python (version 3.10).

## 3 Results

### 3.1 Clinical characteristics

We included 36 controls (26 male and 10 female participants) and 35 patients with VaD (20 male and 15 female participants). Significantly higher degrees of severity of PVH and DWMH were observed in the VaD group (*p* < 0.05), and no significant differences were found in age, sex, BMI, education, and VRFs (except hypertension) between the two groups (Table 1).

**Table 1:** Demographic and clinical characteristics of participants.

	VaD *n* = 35	Controls *n* = 36	*p* value
Female (%)^b^	15 (42.86)	10 (27.8)	0.184
Age (years)^c^	64.1 (7.1)	62.1 (5.1)	0.262
Education (years)^c^	10.8 (3.6)	11.6 (3.1)	0.356
BMI^a^	24.46 (3.21)	23.45 (2.71)	0.155
Number of VRFs^a^	1.8 (0.9)	1.9 (1.0)	0.790
Smoking (%)^b^	10 (28.57)	15 (41.67)	0.248
DM (%)^b^	11 (31.42)	12 (33.34)	0.864
Hypertension (%)^b^	30 (85.71)	20 (55.56)	0.005*
Hyperlipidemia (%)^b^	11 (31.42)	17 (47.22)	0.173
CVD (%)^b^	2 (5.71)	4 (11.12)	0.414
DWMH^c^	2.6 (0.4)	1.6 (0.8)	0.000*
PVH^c^	2.3 (0.8)	1.9(0.9)	0.041*

VaD, vascular depression; BMI, body mass index; CVD, cardiovascular disease; PVH, periventricular white matter hypertension; DWMH, deep white matter hypertension. Data are presented as the mean (standard deviation [SD]) or number of participants in each group (% of total). ^*^p < 0.05.

a Independent-samples *t* test.

b Chi-squared test.

c Mann-Whitney U test.

Neuropsychological assessment revealed significantly higher HAMD-17 and HAMA-14 scores in the VaD group than in the controls (*p* < 0.01) (Table 2). Significant cognitive impairment was observed in the VaD group (*p* < 0.01, MMSE; *p* = 0.023; TMT-B score, *p* = 0.036) (Table 2).

**Table 2:** Neuropsychological assessment of participant.

	VaD *n* = 35	Controls *n* = 36	*p* value
HAMD-17^c^	15.6 (5.25)	3.2 (2.25)	0.000*
HAMA-14^c^	10.1 (4.98)	3.7 (2.55)	0.001*
TMT-A (s)^c^	59.21 (25.84)	83.63 (43.81)	0.023*
TMT-B (s)^c^	106.11 (74.48)	140.91 (85.11)	0.036*
MMSE^c^	25.1 (2.41)	26.5 (2.04)	0.011*

HAMD-17, hamilton depression scale 17-item; HAMA-14, hamilton anxiety scale 14-item; MMSE, mini-mental state examination; TMT-A/B, trail making test A/B. Data are presented as the mean (standard deviation [SD]) ^*^p < 0.05. ^c^Mann-Whitney U test.

### 3.2 Protein identification by whole-proteome analysis

**Figure 1:**
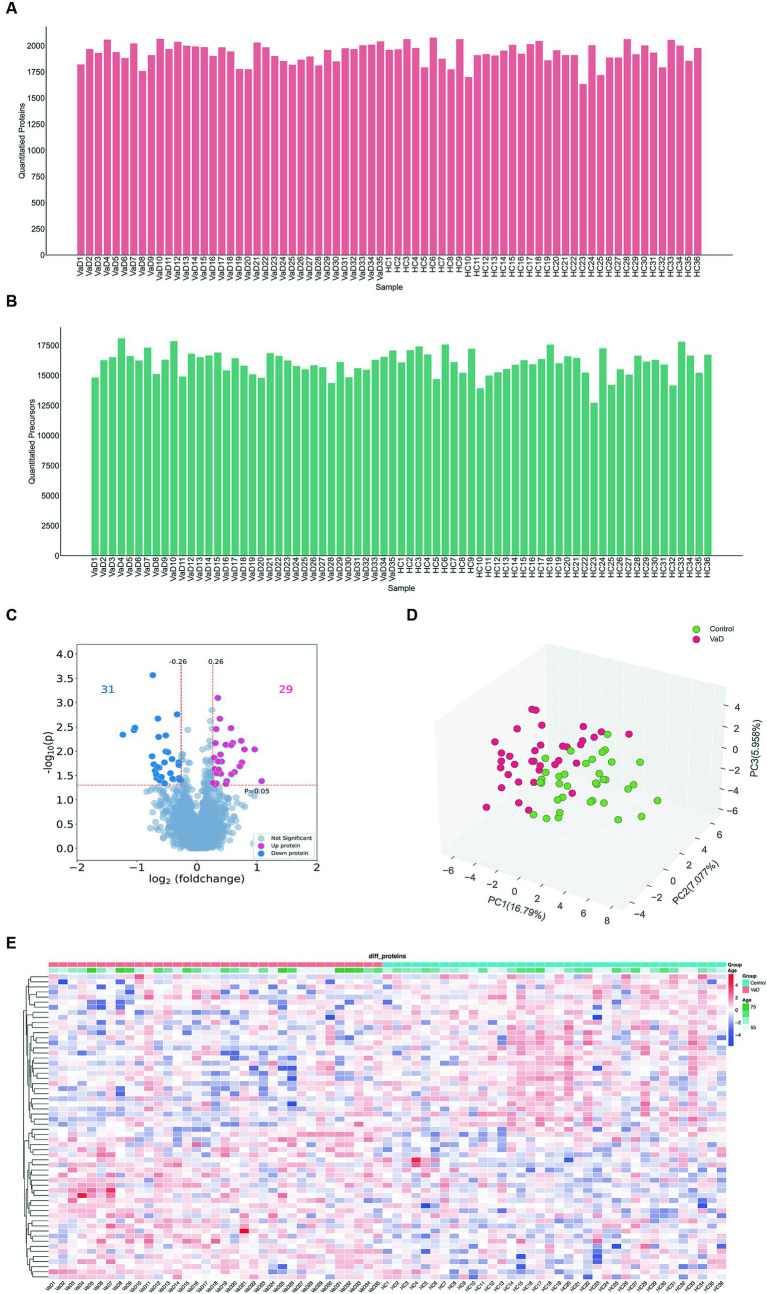
Analysis of whole proteomics data. **(A,B)** Quantification of the quantity distribution of proteins and peptides. **(C)** Volcano plot of all identified proteins (|log2FC| ≥ 0.26, *p* ≤ 0.05) (up-graded shown in red and down-graded shown in blue). **(D)** Heatmap showing 60 significantly different proteins in VaD and control groups. **(E)** PCA plots. Each point represents an individual protein. VaD: vascular depression.

We analyzed 71 samples from the entire cohort using DIA and quantified 2,351 proteins and 23,769 peptides (Figures 1A,B). An overview of the dataset quality is presented in Supplementary Figure S1. Serum proteomic analysis revealed 60 significantly altered proteins in patients with VaD, of which 29 were significantly up-regulated, and 31 were significantly downregulated compared to the controls (Figures 1C,D). All samples’ principal component analysis (PCA) showed that VaD and controls could be differentiated effectively with the 60 DEPs (Figure 1E). The whole-proteome expression analysis is shown in Supplementary Figure S2.

### 3.3 Functional enrichment and PPI network analysis of DEPs

**Figure 2:**
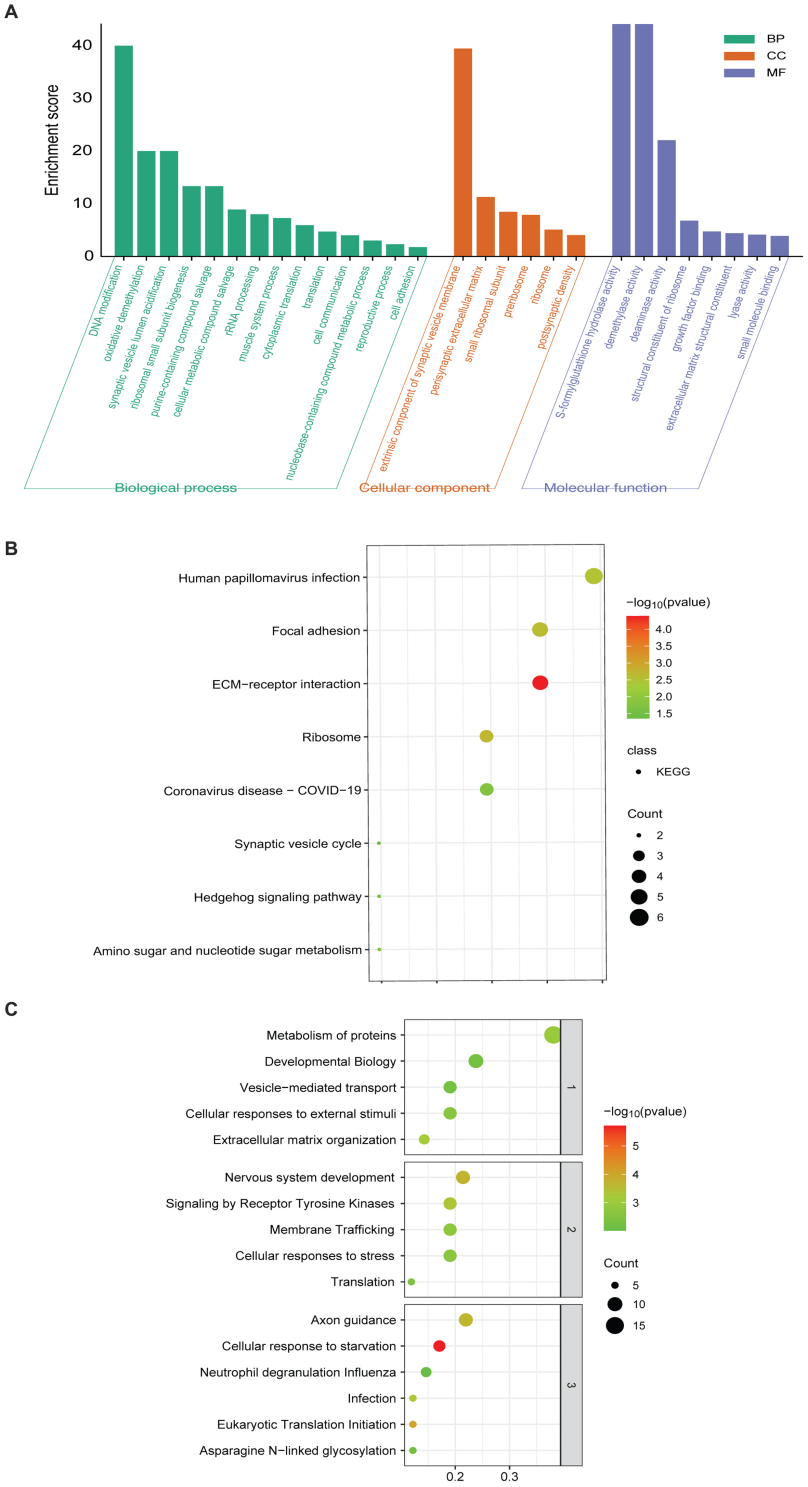
Pathway enriched analyses and PPI network of DEPs. **(A)** GO-based enrichment analysis of DEPs (two-sided hypergeometric test, *p ≤* 0.05), GO terms were sorted by *p*-value, and the top 5 terms of each category were displayed. **(B)** KEGG-based enrichment analysis of DEPs (two-sided hypergeometric test, *p* ≤ 0.05), KEGG terms were sorted by *p*-value, and the top 5 terms were displayed. **(C)** Reactome-based enrichment analysis of DEPs (*p-adj* < 0.05). The different biological levels sorted Reactome terms, and the top 5 terms of each level were displayed.

GO enrichment analysis showed that the 60 DEPs were associated with 14 distinct biological processes (BP) and 8 molecular functions (MF). The most enriched terms of cellular components (CC) were related to extrinsic component of synaptic vesicle membrane and perisynaptic extracellular matrix (Figure 2A). KEGG analyses revealed that the enriched pathways included the synaptic vesicle cycle, extracellular matrix (ECM)-receptor interaction, and focal adhesion (Figure 2B). The top 10 enriched Reactome pathways were presented, involving vesicle-mediated transport, nervous system development, and axon guidance (Figure 2C).

**Figure 3:**
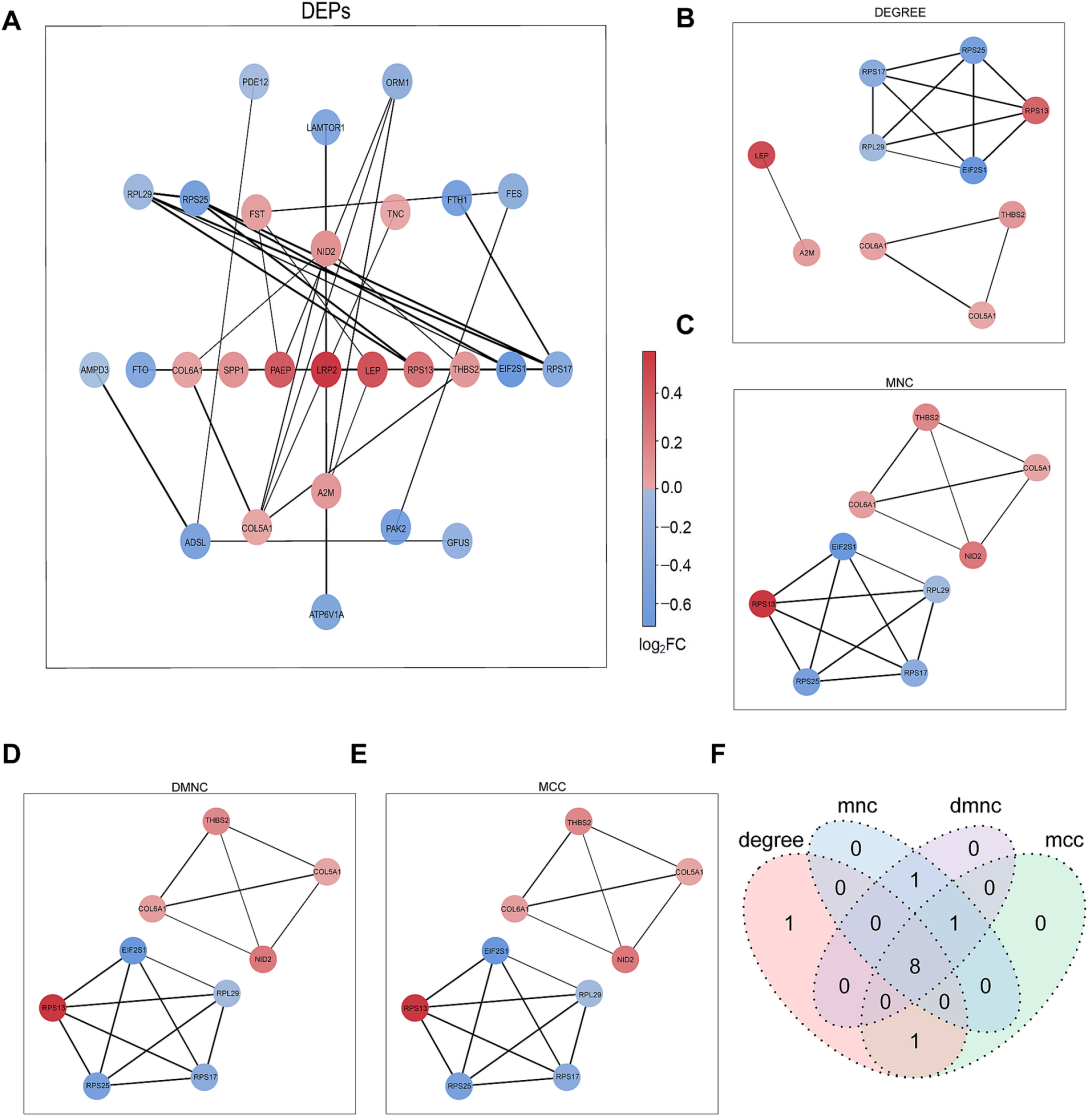
PPI network analysis of DEPs. **(A)** Network graph depicting the correlation of proteins derived from DEPs. **(B–E)** The hub proteins were calculated under **(B)** DEGREE, **(C)** MNC, **(D)** DMNC, and **(E)** MCC. **(F)** The Venn diagram is based on cross-analysis under four algorithms (DEGREE, DMNC, MCC, and MNC). Circles indicated the gene symbol of protein, in which red and blue indicated up-regulated and down-regulated proteins, respectively. The thickness of the lines represented the strength of the interaction. The darkness of the colors represented the magnitude of the discrepancy.

The PPI network of DEPs was constructed using the STRING database (Figure 3A). The top nine proteins in the four algorithms (DEGREE, DMNC, MCC, and MNC) were calculated and exported as hub proteins (Figures 3B–E). Moreover, eukaryotic translation initiation factor 2 subunit 1(IF2A), collagen alpha-1(VI) chain (COL6A1), collagen alpha-1(V) chain (COL5A1), thrombospondin-2 (THBS2), ribosomal protein L29 (RPL29), ribosomal protein S13 (RPS13), ribosomal protein S25 (RPS25), ribosomal protein S25 (RPS17), and Nidogen-2 (NID2) were identified as hub proteins (Figure 3F).

### 3.4 Selection and development of the model

**Figure 4:**
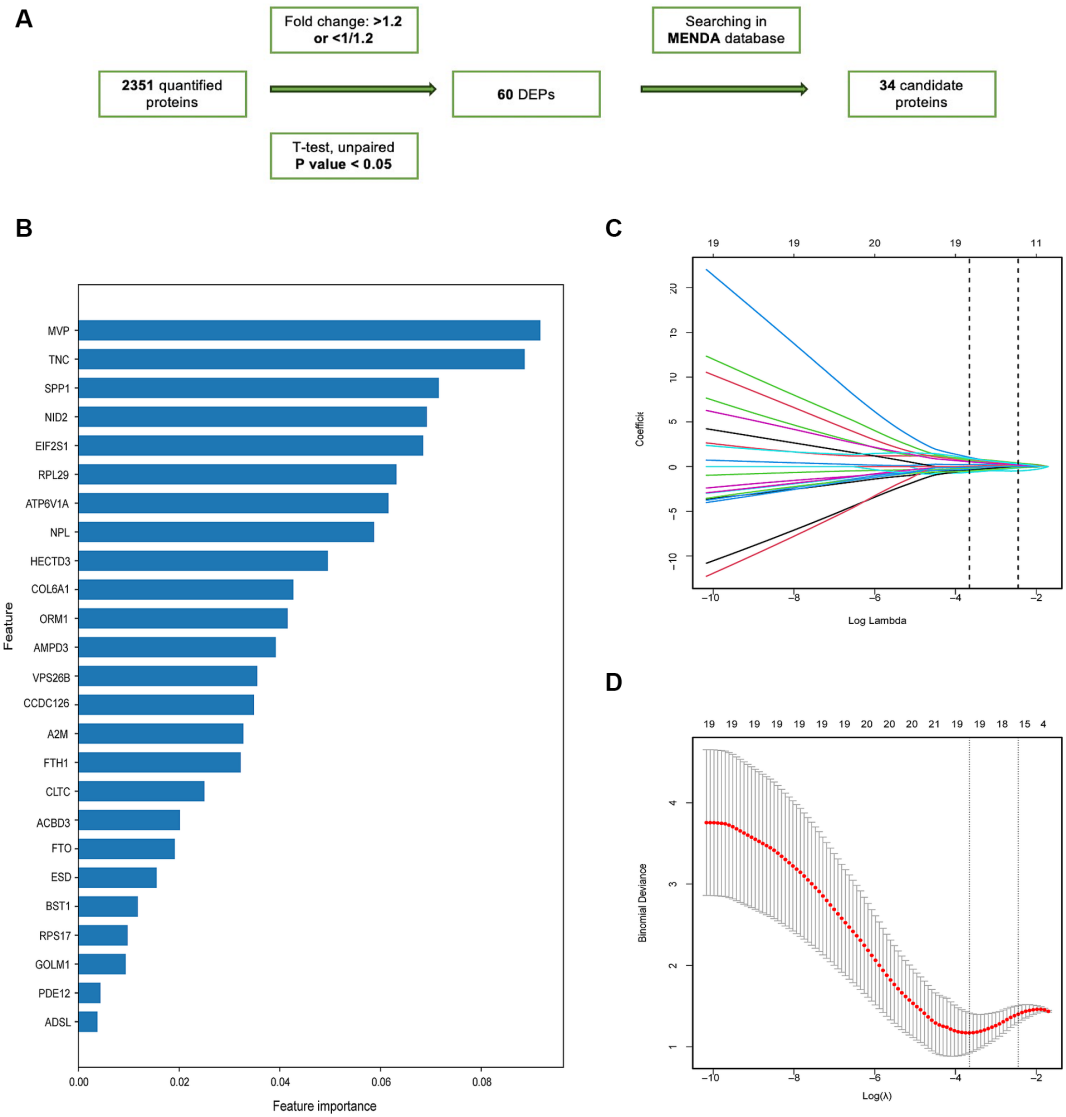
The screening of candidate proteins for VaD was identified by machine learning. **(A)** Flow chart of the screening of the candidate proteins. **(B)** Feature importance map of proteins. The top 25 candidate proteins were selected by feature importance in XGBoosting analysis. **(C)** LASSO coefficient profiles of 25 proteins. **(D)** The relational graph between fitting error and log (λ). Dotted vertical lines were drawn at the optimal values using the minimum criteria (min criteria) and the 1 standard error of the minimum criteria (1se criteria). λ value was chosen when the fitting error was minimal.

Based on a previously described computational pipeline, we selected the important proteomic variables as Figure 4A (Shu et al., 2020). 34 proteins were selected from the DEPs for subsequent analysis based on their intersection with the depression proteomic database (MENDA)^4^ (see Supplementary Table S1 for detailed information). The importance matrix plot for the XGBoost method revealed the top 25 variables contributing to the prediction in the training cohort (Figure 4B). LASSO regression analysis further filtered the protein features that were considered candidate biomarkers for predicting VaD (Figures 4C,D). Consequently, 19 candidate proteins were selected for model development. Using five-fold cross-validation, and following the principle of a random combination of less than or equal to 5 proteins, we identified the combination with the optimal AUC value, including HECT domain E3 ubiquitin protein ligase 3 (HECTD3), NID2, FTO alpha-ketoglutarate-dependent dioxygenase (FTO), Golgi membrane protein 1 (GOLM1), and N-acetylneuraminate lyase (NPL).

### 3.5 Model evaluation

**Figure 5:**
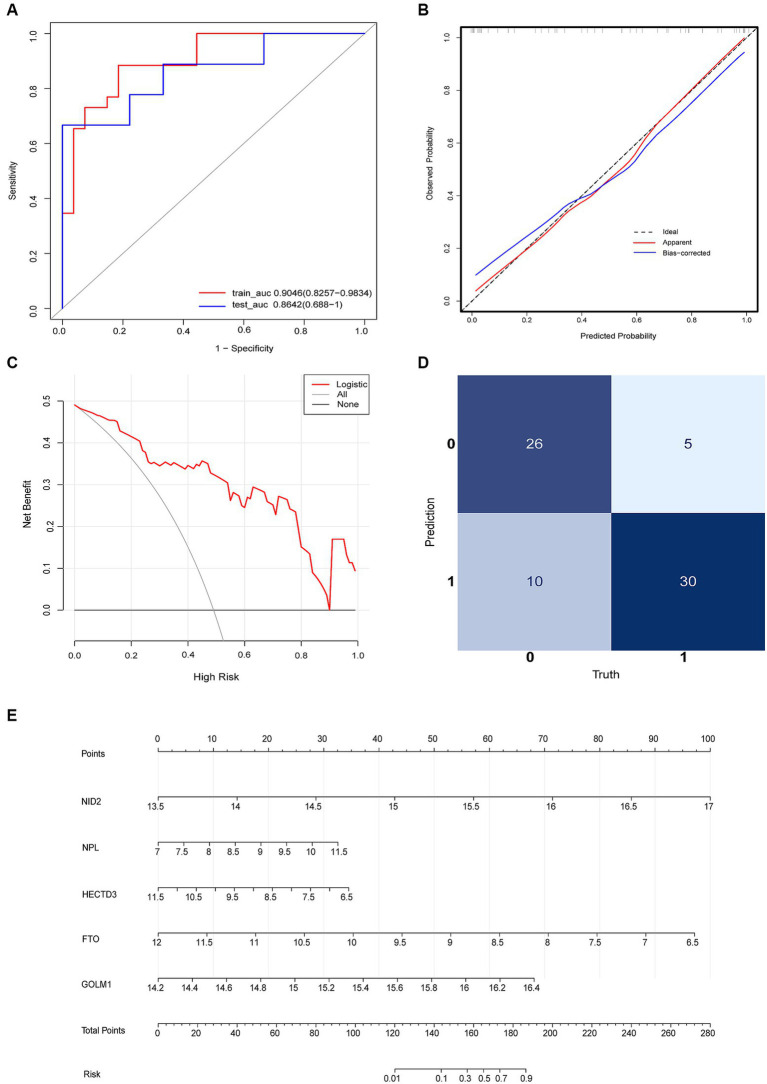
Logistic regression model evaluation. **(A)** The receiver operating characteristic curve (ROC). **(B)** The calibration curve and **(C)** DCA showed the model had a promising clinical performance. **(D)** Confusion matrix. **(E)** The nomogram with the relative expression level of HECTD3, NID2, FTO, GOLM1, and NPL for predicting the risk of VaD.

The diagnostic model with satisfactory discrimination was constructed. The AUC [95% confidence interval (CI)] for the training and testing cohorts were 0.9046 (0.8257–0.9834) and 0.8643 (0.688–1), respectively (Figure 5A). A calibration curve was constructed to evaluate the reliability of the machine learning strategy, which showed good performance compared with the ideal model (Figure 5B). In addition, the model showed promising clinical performance according to decision curve analysis (DCA) (Figure 5C). The confusion matrix showed excellent efficacy in differentiating between groups with an accuracy of 78.9% (56/71) (Figure 5D). This model had a sensitivity of 85.7% (30/35) and a specificity of 72.2% (26/36). The positive prediction values were 75.0% (30/40) and the negative were 83.9% (26/31). The nomogram visualized the risk prediction model based on the serum relative expression levels of the 5 identified proteins (Figure 5E).

**Figure 6:**
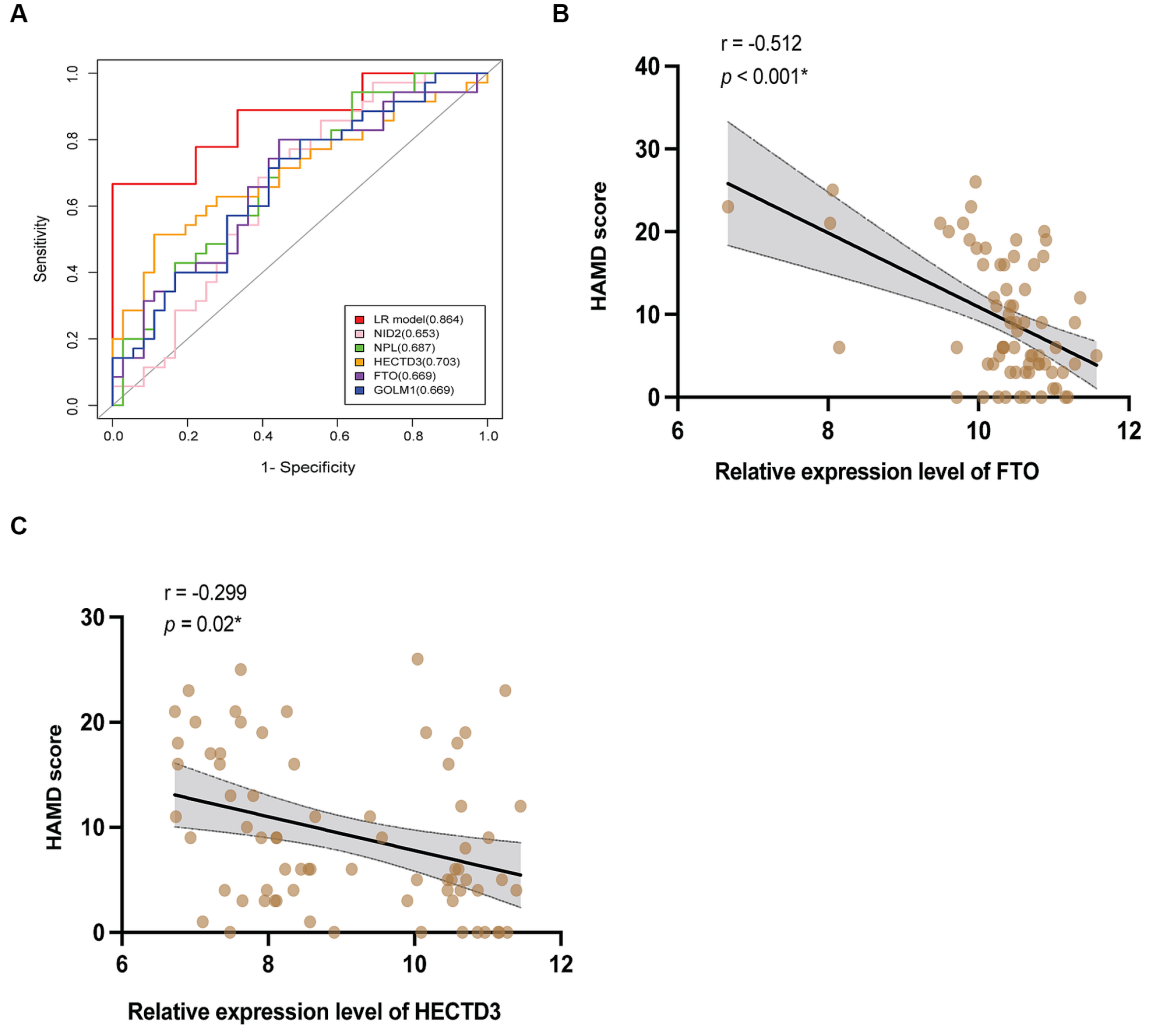
The clinical values of the 5 proteins. **(A)** The ROC curves of HECTD3, FTO, GOLM1, NPL, and NID2, compared to the combination. **(B,C)** The results of correlation analysis of HECTD3 and FTO with HAMD-17 score.

Based on this algorithm, ROC curves for the 5 individual proteins were plotted and showed less favorable diagnostic performance than the biomarkers combination (Figure 6A). Multivariable logistic regression showed that the high relative expression level of NID2, GOLM1, and NPL, and the low expression level of HECTD3, and FTO were associated with a high risk of VaD (see Supplementary material). After controlling the confounding factors, the level of HECTD3 and FTO demonstrated moderate correlations with HAMD-17 scores (Figures 6B,C). No significant correlation was found between the other three proteins and the severity of depressive symptoms.

## 4 Discussion

Depression is one of the most important public health issues in the older adult population. Approximately 14% of individuals aged over 55 years exhibit depressive syndrome, with only 2% having MDD (Kok and Reynolds, 2017). It is important to identify biomarkers that can monitor and predict the development of this disease and understand its pathogenesis. Our study presents the first comprehensive serum proteomic analysis of VaD and developed a proteomics-based diagnostic model with machine learning, including HECTD3, NID2, FTO, GOLM1, and NPL.

Current research on protein markers of VaD is limited. The anti-aging gene Sirtuin 1 (SIRT1) was confirmed to be a depression and stroke-related gene (Martins, 2016; Li et al., 2022). Sirtuin 1 is a critical nuclear deacetylase that participates in regulating the transcription of various transcription factors and cellular signal transduction proteins, involving inflammation, neurogenesis, glucose/cholesterol metabolism, and amyloidosis (Lu et al., 2018; Man et al., 2019; Ministrini et al., 2021). Sirtuin 1 plays a crucial role in the development of vascular and cerebrovascular diseases. Previous studies have implicated the hippocampal SIRT1 pathway in chronic stress-induced depression-related phenotypes and abnormal dendritic atrophy (Abe-Higuchi et al., 2016). Notably, it has been reported that Sirtuin 1 exerted the degradation of FTO, which was significantly decreased in VaD and negatively associated with depressive symptoms in our study (Liu et al., 2020). The potential of Sirtuin 1 as a marker for VaD deserves validation in more research.

Interestingly, the function of the proteins contained in the biomarkers panel and the signaling pathways involved have been implicated in multiple mechanisms of VaD.

FTO is a demethylase of *N*^6^-methyladenosine (m^6^A) enriched in brain neurons, playing an important role in the mechanism of depression (Jia et al., 2011; Mitsuhashi and Nagy, 2023). Its expression level and demethylase activity are severely affected after ischemic injury (Jia et al., 2011; Xu H. et al., 2020). It’s reported that the downregulation of FTO in the anterior cingulate cortex (ACC) by modulating matrix metalloproteinase-9 (MMP-9) mRNA methylation participates in anxiety- and depression-like behaviors in neuropathic pain (Wang et al., 2022). In addition, a recent study has confirmed the neuroprotective effects of FTO in acute ischaemic injury, regulating white and grey matter damage and ameliorating cognitive decline and depressive-like behavior after stroke (Chokkalla et al., 2023). FTO is suggested to be a novel molecule mediating neurotransmitter transmission, neuroplasticity, neurogenesis, and memory formation (Li et al., 2017; Yu et al., 2018). Previous studies have shown that FTO contributes to the BDNF processing, and up-regulates the BDNF–TrkB pathway in the hippocampus through m6A modification (Spychala and Rüther, 2019; Xu K. et al., 2020; Chang et al., 2023). It is believed that the pathophysiology of MDD is specifically associated with a decrease in hippocampal BDNF activity and function. Numerous studies as well as the meta-analysis show that depressed populations have lower serum and plasma levels of BDNF, which was considered a promising biomarker for depression (Molendijk et al., 2014; Nedic Erjavec et al., 2021). Polymorphisms of the FTO gene have been linked to depression and metabolic syndromes (Liguori et al., 2014; Rivera et al., 2017; Zarza-Rebollo et al., 2021). Our findings reinforce previous evidence and suggest that abnormal expression of the FTO protein and related pathways also play an important role in susceptibility to VaD.

Proteomic studies have identified altered expression of inflammatory proteins in individuals with LLD, particularly emphasizing the dysregulation of inflammation and immune responses (Diniz et al., 2016; Silva-Costa et al., 2022). It is evidenced by the vulnerable but significant correlation between the onset and development of depression and elevated levels of inflammatory markers such as CRP, TNF-α, IL-6, and IL-1 (Hacimusalar and Eşel, 2017). It has been documented that HECTD3 participates in protein ubiquitination and modifies a range of substrate proteins, is exposed to multiple regulatory mechanisms, and is essential for cellular functions like immune response, neuroinflammation, and apoptosis (Cho et al., 2019; Jiang et al., 2020). HECTD3 promotes NLRP3 inflammasome and pyroptosis, thereby exacerbating diabetes-related cognitive impairment by stabilizing MALT1 and regulating the JNK pathway (Ruan et al., 2022). Also, HECTD3 inhibits Stat1 to reduce the secretion of pro-inflammatory factors (Rangrez et al., 2020). However, the mechanisms of HECTD3 mediating the inflammation in VaD remain unclear. In addition, GOLM1 is considered involved in immunoregulation and inflammation (Pu et al., 2021). No studies have yet discovered its underlying association with depression. However, it was reported that genetic variation in GOLM1 is associated with reduced gray matter volume in the left frontal gyrus in Alzheimer’s disease (AD) (Inkster et al., 2012). Diffusion tensor imaging studies revealed microstructural damage to white matter tracts connecting the prefrontal cortex in geriatric depression, which was related to executive dysfunction (He et al., 2021).

NID2, identified as a hub protein in the PPI network analysis, is a basement membrane glycoprotein. It participated in cell-extracellular matrix interactions, essential for preserving the contractile characteristics of smooth muscle cells in blood vessels and regulating vascular homeostasis (Mao et al., 2021). Because of the links between risk factors for endothelial damage (i.e., hyperglycemia, hypertension, metabolic disorders) and depression, endothelial dysfunction may increase the vulnerability to VaD (Virtanen et al., 2017). NPL is a metabolic protein, involved in the N-glycolylneuraminic acid (Neu5Gc) degradation pathway (Da Silva et al., 2023). Overexpression of Neu5Gc in the brain resulted in abnormal axon myelination and impaired memory (Naito-Matsui et al., 2017). Overall, the alterations of protein levels in peripheral blood could reflect the pathological damage and be a potential diagnostic indicator for VaD.

Based on the bioinformatics analysis, we confirmed previous findings that depression in older adults involves multiple biological processes related to vesicle-mediated transport, DNA modification, oxidative demethylation, and metabolism. Moreover, our study revealed that the synaptic vesicle cycle was dysregulated in VaD. In presynaptic terminal biology, the synaptic vesicle cycle plays a pivotal role in mediating a series of events that allow chemical neurotransmission between functionally linked neurons, which have been connected to several different neuropsychiatric conditions, such as bipolar disorder, depression, and dementia (Sudhof, 2004; Si et al., 2018; Wu et al., 2022; Tang et al., 2023). Chronically reduced cerebral blood flow induces ischemia and hypoxia, affecting the function of neurons and synapses and increasing vulnerability to stress and depression (Nobler et al., 1999; Yan et al., 2020; van Aalst et al., 2021). Our results indicate that abnormalities in the synaptic vesicle cycle and abnormal modulation of peri- and post-synaptic adhesion molecules are related to decreased synaptic plasticity, demonstrating that this could be a mechanism underlying VaD.

### 4.1 Limitations

The limitations of the study should be taken into account. Firstly, there was the probability of type I statistical error, and the results have yet to be independently replicated. The identified biomarkers panel needs verification and validation in external cohorts. In addition, mass spectrometry-based proteomics is inherently characterized by the specificity of detection and quantification, but relatively lower sensitivity in detecting lower abundance proteins, compared to ELISA and Western Blot, which can amplify the signal in a cascade (Bader et al., 2023). Also, many parameters characterize VaD, and we could not assess the impact of clinical factors (hypertension, WMH) on proteomic changes in VaD. Hypertension is associated with more severe white matter damage, leading to the susceptibility to VaD. It requires further subgroup analyses in larger samples to assess the role of these confounding factors in VaD. Nevertheless, the main strengths of our study are the development of the proteomics-based model with favorable effectiveness and the relatively large antidepressant-free sample source. The findings of participants with mild to moderate depressive symptoms are beneficial in the early diagnosis of VaD and deserve more research in MDD patients.

## 5 Conclusion

In conclusion, this study provides an indispensable proteomics resource to gain a better understanding of VaD, unravel its underlying pathogenesis, and identify a promising panel of biomarkers with the proteomics-based model in early screening for VaD. We speculate that some significantly altered proteins and related pathways identified in this work may be potential therapeutic targets for VaD.

## Data availability statement

The mass spectrometry proteomics data have been deposited to the ProteomeXchange Consortium (http://proteomecentral.proteomexchange.org) via the iProX partner repository with the dataset identifier PXD047302.

## Ethics statement

The studies involving humans were approved by Institutional Ethics Committee of Zhongnan Hospital, Wuhan University. The studies were conducted in accordance with the local legislation and institutional requirements. The participants provided their written informed consent to participate in this study.

## Author contributions

LL: Conceptualization, Data curation, Formal analysis, Investigation, Visualization, Writing – original draft. SP: Conceptualization, Supervision, Validation, Writing – review & editing. RZ: Data curation, Writing – original draft. HH: Data curation, Writing – original draft. YY: Methodology, Software, Writing – original draft. BX: Methodology, Software, Writing – original draft. JZ: Funding acquisition, Project administration, Resources, Writing – review & editing.

## Footnotes

^1^www.geneontology.org

^2^http://www.genome.jp/kegg/

^3^https://reactome.org

^4^http://menda.cqmu.edu.cn
