# Supplementary material for: Serum proteomic biomarker investigation of vascular depression using data-independent acquisition: a pilot study
Source: Front Aging Neurosci. 2024 Feb 7;16:1341374. doi: 10.3389/fnagi.2024.1341374 (PMC10879412; doi:10.3389/fnagi.2024.1341374)
Supplement: Supplementary file 2 [file Data_Sheet_2.docx]

Supplementary Material

Serum proteomic biomarker investigation of vascular depression using data-independent acquisition: a pilot study

**Liuyi Lan^1†^, Sisi Peng^2†^, Ran Zhang^1^, Haoying He^1^, Yong Yang^3^, Bing Xi^3^, Junjian Zhang^1^***

*** Correspondence:** Junjian Zhang; [zhangjj@whu.edu.cn](mailto:zhangjj@whu.edu.cn)

# Supplementary Figures and Tables

## Supplementary Figures

**Supplementary Figure 1.** Quality control. **(A)** Coverage of protein sequence; **(B)** Protein amount distribution graph; **(C)** Distribution of missed cut sites; (D) Peptide number distribution graph; **(E)** Peptide length distribution graph.

**Supplementary Figure 2.** Protein expression analysis. **(A)** Quantitative overview plot of all proteins with quantitative values of z-score; **(B)** Volcano plot of differential proteins with 1.5-fold change; **(C)** Volcano plot of differential proteins with 2.0-fold change. Red indicates up-regulated proteins, and blue indicates down-regulated proteins; **(D**) Differential protein profiles at different radio bord; **(E)** All protein principal component analysis (PCA) plot.

## Supplementary Tables

| **Supplementary Table 1.** 34 candidate proteins were obtained after intersecting with the depression database (MENDA) (http://menda.cqmu.edu.cn） | | | | |
| --- | --- | --- | --- | --- |
| **Candidate proteins** | | | **Menda database** | |
| **Accession** | **Gene name** | **Description** | **Organism** | **Tissue** |
| P01023 | A2M | Alpha-2-macroglobulin | Human | serum, Cerebrospinal fluid |
| P02763 | ORM1 | Alpha-1-acid glycoprotein 1 | Human | serum, Cerebrospinal fluid |
| P02794 | FTH1 | Ferritin heavy chain | Human | Subgenual anterior cingulate cortex |
| P05198 | EIF2S1 | Eukaryotic translation initiation factor 2 subunit 1 | Chronic mild stress model | Hypothalamus,Cerebrospinal fluid |
| P08708 | RPS17 | 40S ribosomal protein S17 | Chronic mild stress model | Hippocampus |
| P10451 | SPP1 | Osteopontin | Chronic mild stress model | Urine |
| P10768 | ESD | S-formylglutathione hydrolase | Human | Anterior prefrontal cortex |
| P12109 | COL6A1 | Collagen alpha-1(VI) chain | Human | Cerebrospinal fluid |
| P24821 | TNC | Tenascin | Chronic mild stress model | Hippocampus, Prefrontal cortex |
| P30566 | ADSL | Adenylosuccinate lyase | Chronic mild stress model | Cerebellum |
| P38606 | ATP6V1A | V-type proton ATPase catalytic subunit A | Human | Anterior prefrontal cortex, Dorsolateral prefrontal cortex |
| P47914 | RPL29 | 60S ribosomal protein L29 | Myocardial infarction induced depression model | Hippocampus,Heart |
| P62277 | RPS13 | 40S ribosomal protein S13 | Social defeat model | Faece |
| P62851 | RPS25 | 40S ribosomal protein S25 | Chronic mild stress model | Cerebrospinal fluid |
| Q00610 | CLTC | Clathrin heavy chain 1 | Human | Anterior prefrontal cortex, Peripheral blood mononuclear cell |
| Q01432 | AMPD3 | AMP deaminase 3 | Chronic mild stress model | Prefrontal cortex, Ventral hippocampus |
| Q10588 | BST1 | ADP-ribosyl cyclase/cyclic ADP-ribose hydrolase 2 | Human | Plasma |
| Q13630 | GFUS | GDP-L-fucose synthase | Social defeat model | Faece |
| Q14112 | NID2 | Nidogen-2 | Chronic mild stress model | Hippocampus |
| Q14764 | MVP | Major vault protein | Chronic mild stress model | Cerebellum |
| Q4G0F5 | VPS26B | Vacuolar protein sorting-associated protein 26B | Chronic social isolation induced depression model | Prefrontal cortex |
| Q5T447 | HECTD3 | E3 ubiquitin-protein ligase HECTD3 | Corticosterone induced depression model | Hippocampus,Hypothalamus |
| Q6IAA8 | LAMTOR1 | Ragulator complex protein LAMTOR1 | Chronic mild stress model | Hippocampus |
| Q6L8Q7 | PDE12 | 2',5'-phosphodiesterase 12 | Chronic mild stress model | Hippocampus |
| Q8NBJ4 | GOLM1 | Golgi membrane protein 1 | Human | Cerebrospinal fluid |
| Q96EE4 | CCDC126 | Coiled-coil domain-containing protein 126 | Human | Plasma |
| Q9BXD5 | NPL | N-acetylneuraminate lyase | Chronic mild stress model | Striatum |
| Q9C0B1 | FTO | Alpha-ketoglutarate-dependent dioxygenase FTO | Global cerebral ischemia induced depression model | Hippocampus |
| Q9H3P7 | ACBD3 | Golgi resident protein GCP60 | Chronic mild stress model | Hippocampus |
| P61163 | ACTR1A | Alpha-centractin OS=Homo sapiens OX=9606 GN=ACTR1A PE=1 SV=1 | Chronic mild stress model | Hippocampus |
| P19883 | FST | Follistatin OS=Homo sapiens OX=9606 GN=FST PE=1 SV=2 | Human | Serum |
| P48729 | CSNK1A1 | Casein kinase I isoform alpha OS=Homo sapiens OX=9606 GN=CSNK1A1 PE=1 SV=2 | Human | Subgenual anterior cingulate cortex |
| P62166 | NCS1 | Neuronal calcium sensor 1 OS=Homo sapiens OX=9606 GN=NCS1 PE=1 SV=2 | Chronic mild stress model | Hippocampus |
| P98164 | LRP2 | Low-density lipoprotein receptor-related protein 2 OS=Homo sapiens OX=9606 GN=LRP2 PE=1 SV=3 | Social defeat model | Faece |

# Supplementary technical support

**The model parameters we used:**

Lasso：family = 'binomial',type.measure = 'deviance',nfolds = 10,lambda = 0.03397

XGBoost: select default parameters.

XGBClassifier(base_score=0.5, booster='gbtree', callbacks=None,

colsample_bylevel=1, colsample_bynode=1, colsample_bytree=1,

early_stopping_rounds=None, enable_categorical=False,

eval_metric=None, feature_types=None, gamma=0, gpu_id=-1,

grow_policy='depthwise', importance_type=None,

interaction_constraints='', learning_rate=0.300000012,

max_bin=256, max_cat_threshold=64, max_cat_to_onehot=4,

max_delta_step=0, max_depth=6, max_leaves=0, min_child_weight=1,

missing=nan, monotone_constraints='()', n_estimators=100,

n_jobs=0, num_parallel_tree=1, predictor='auto', random_state=0, ...)

**The packages are as follows:**

Using ‘from sklearn.metrics import roc_curve’. ‘sklearn.metrics’ is a module in the scikit-learn library, specifically used to provide various evaluation indicators (metrics) to evaluate the performance of machine learning models. ‘roc_curve’ is a function in the scikit-learn library, used to calculate the Receiver Operating Characteristic curve (ROC curve). The ROC curve is a graphical tool used to evaluate the performance of a binary classification model. This curve takes the False Positive Rate (FPR) as the horizontal axis and the True Positive Rate (TPR, also known as the recall rate) as the vertical axis, showing the performance of the model under different thresholds.

The reference webpage is as follows:

<https://scikit-learn.org/stable/modules/generated/sklearn.metrics.roc_curve.html#sklearn.metrics.roc_curve>
