# Supplementary figures and images for: Serum proteomic biomarker investigation of vascular depression using data-independent acquisition: a pilot study
Source: Front Aging Neurosci. 2024 Feb 7;16:1341374. doi: 10.3389/fnagi.2024.1341374 (PMC10879412; doi:10.3389/fnagi.2024.1341374)

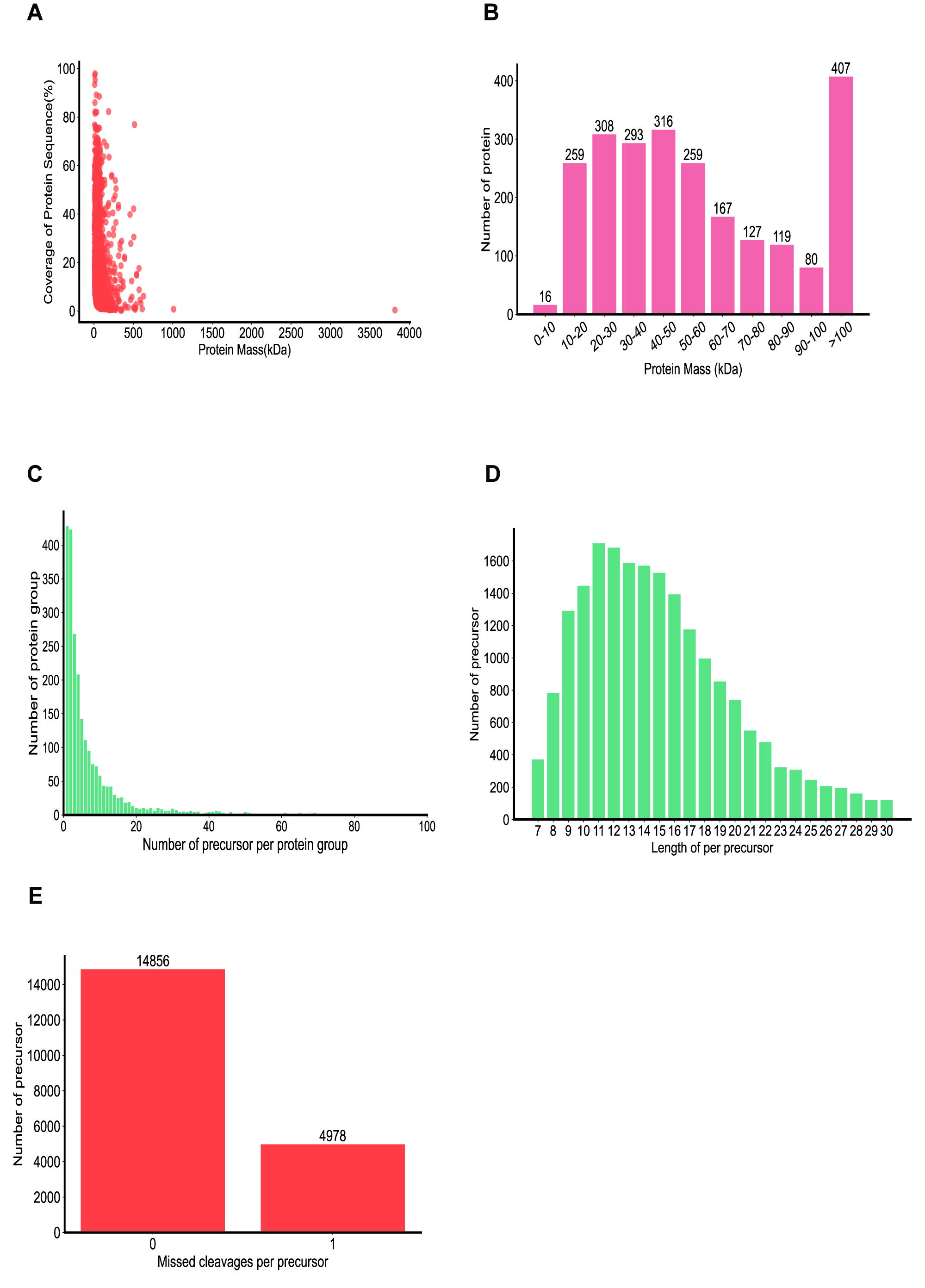

Supplement: Supplementary file 3 [file Image_1.TIF]

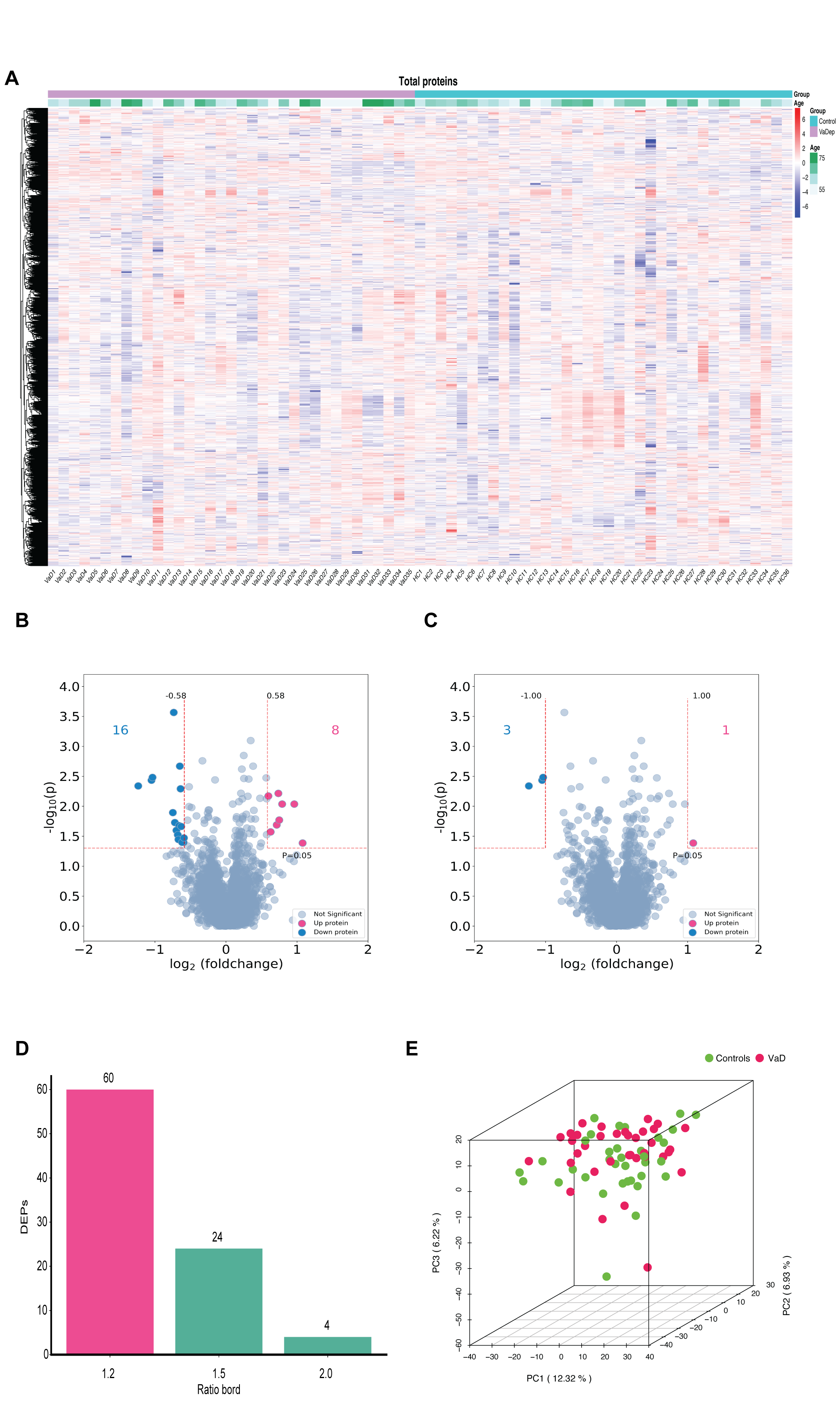

Supplement: Supplementary file 4 [file Image_2.TIF]
